# Discovery and functional characterization of novel programmed death-ligand 1 monoclonal antibodies

**DOI:** 10.55730/1300-0152.2974

**Published:** 2026-05-31

**Authors:** Nurşah ERSEZEN, Maide ŞEKER, Arzu AYSAN, Durmuş AKDOĞAN, Mehmet Ender AVCI, Aslı KURDEN PEKMEZCİ, Sibel KALYONCU UZUNLAR, Mehmet İNAN, Tamer YAĞCI

**Affiliations:** 1 Department of Molecular Biology and Genetics, Faculty of Sciences, Gebze Technical University, Kocaeli, Turkiye; 2 Central Research Laboratory, Gebze Technical University, Kocaeli, Turkiye; 3 İzmir Biomedicine and Genome Center, İzmir, Turkiye; 4 Dokuz Eylül University, İzmir, Turkiye; 5 İzmir International Biomedicine and Genome Institute, Dokuz Eylül University, İzmir, Turkiye

**Keywords:** PD-L1, immunotherapy, immune checkpoint protein, monoclonal antibody, hybridoma technology

## Abstract

**Background/aim:**

Targeted therapies with monoclonal antibodies provide cancer patients with better prognosis and disease-free survival. The blockade of immune checkpoints, including programmed cell death protein-1 (PD-1) and its ligand PD-L1, with monoclonal antibodies may boost immune responses against tumors and is regarded as an effective strategy in cancer immunotherapy. We describe the generation of anti-PD-L1 monoclonal antibodies with high affinity and specificity, and we assess their potential for therapeutic use in cancer.

**Materials and methods:**

Hybridomas were selected for PD-L1 specificity and cross-reactivity with other immune checkpoint proteins and PD-L1 orthologs using indirect ELISA. Immunofluorescence and Western blotting assays were conducted for further characterization of the antibodies. The affinities of the antibodies for PD-L1 were determined using surface plasmon resonance. Receptor blocking activities were examined through competitive ELISA and cell-based luciferase reporter assays. Sequences of variable regions of the selected antibodies were determined by Sanger sequencing and subjected to BLAST analysis.

**Results:**

A total of 25 PD-L1-specific monoclonal antibodies were generated. While most clones reacted with PD-L1 from cynomolgus monkeys, none of the antibodies displayed cross-reactivity with other checkpoint proteins. Immunofluorescence assays showed that the selected clones stained PD-L1-expressing cell membranes specifically, but not those of PD-L1-negative cells. Western blotting revealed that most of the clones recognized both glycosylated and nonglycosylated PD-L1, and a few reacted with the glycosylated form only. Only two clones with subnanomolar affinity for human PD-L1 were effective at blocking PD-1/PD-L1 and CD80/PD-L1 interactions. Sequence analysis of their variable regions revealed their unique specificity.

**Conclusion:**

Of the 25 monoclonal antibodies produced in this study, only one was identified as a potential therapeutic drug candidate thanks to its high capacity for checkpoint blockade and affinity, as well as its unique sequence specificity. These properties are comparable to those of anti-PD-L1 antibodies currently used in clinical practice.

## 1. Introduction

The identification of immune checkpoint pathways has had a profound impact on modern oncology, revealing that tumors can exploit physiological mechanisms of immune tolerance to evade immune-mediated destruction (Pardoll, 2012; Chen and Mellman, 2013). Consequently, immune checkpoint blockade has reshaped the paradigm of cancer therapy, evolving from a novel therapeutic concept into a central component of treatment strategies for various malignancies (Topalian et al., 2012). Among immune regulatory pathways, the interaction between programmed cell death protein-1 (PD-1) and its ligand, programmed death-ligand 1 (PD-L1), is recognized as a pivotal axis of tumor-mediated immune suppression and a key regulator of antitumor immunity (Keir et al., 2008). Under physiological conditions, PD-L1 is expressed on antigen-presenting and peripheral tissue cells, and it binds to PD-1 on activated T cells. This maintains immune homeostasis, limits excessive inflammation, and sustains peripheral tolerance by inactivating T cells (Keir et al., 2008; Gao et al., 2024; Liu et al., 2024). However, many tumor types upregulate PD-L1 expression within the tumor microenvironment, thereby suppressing cytotoxic T-cell activity and facilitating immune escape (Yi et al., 2021; Tufail et al., 2025). Therefore, the therapeutic targeting of PD-1/PD-L1 interaction using monoclonal antibodies has become one of the fundamental strategies in cancer immunotherapy (Pardoll, 2012).

Monoclonal antibodies (mAbs) that target PD-L1 disrupt its interaction with PD-1, resulting in immune-mediated tumor elimination through the restoration of T-cell effector functions (Brahmer et al., 2012; Topalian et al., 2012). Clinically approved anti-PD-L1 antibodies such as atezolizumab, durvalumab, avelumab, and sugemalimab have provided significant benefits for patients with malignant tumors including nonsmall-cell lung cancer (NSCLC), urothelial carcinoma, and triple-negative breast cancer (Fehrenbacher et al., 2016; Antonia et al., 2017; Schmid et al., 2018; Powles et al., 2020; Zhou et al., 2025). However, response rates differ between tumor types and patients, and documented cases of patients with no response or acquired resistance have been linked to various tumor-intrinsic and immune evasion mechanisms (Sharma et al., 2017; Havel et al., 2019). These observations underscore the importance of continued development and detailed characterization of novel anti-PD-L1 antibody candidates with effective binding and blocking properties (Havel et al., 2019). Hybridoma technology is a well-established and reliable method for generating high-affinity monoclonal antibodies (Pedrioli and Oxenius, 2021). However, antibodies produced using this method must undergo rigorous screening to ensure specificity and exclude cross-reactivity with structurally related immune checkpoint molecules (Keir et al., 2008). Although high affinity is often desirable, binding strength alone does not guarantee the functional efficacy of antibodies. Importantly, PD-L1 interacts not only with PD-1 but also with CD80, contributing to additional layers of immune regulation. Therefore, assessing antibody-mediated inhibition across the PD-1/PD-L1 and CD80/PD-L1 axes using biochemical and cell-based systems provides a more comprehensive understanding of the potential of checkpoint blockade (Butte et al., 2007; Brahmer et al., 2012; Topalian et al., 2012). The structural complexity of PD-L1, notably its glycosylation status, which influences epitope accessibility, makes the confirmation of target recognition in cell-based systems essential for evaluating the translational potential of candidate antibodies (Lee et al., 2019; Lu et al., 2025). Collectively, these factors highlight the necessity of an integrated evaluation strategy incorporating specificity profiling, affinity determination, receptor blockade capacity, and functional validation (Rich and Myszka, 2000; Sharma et al., 2017).

In the present study, novel anti-PD-L1 mAbs were discovered using hybridoma technology and subjected to comprehensive characterization. The resulting clones were evaluated for specificity and cross-reactivity against related immune checkpoint molecules, and their binding kinetics were determined by surface plasmon resonance (SPR) analysis. Receptor blockade capacity was assessed using competitive assays targeting both PD-1/PD-L1 and CD80/PD-L1 interactions. Furthermore, the restoration of signaling activity at the cellular level was confirmed by examining the functional inhibition of the PD-1/PD-L1 checkpoint axis in cell-based reporter systems. The results indicate the successful production of high-affinity anti-PD-L1 mAbs that exhibit robust blockade activity, supporting the potential of these mAbs as therapeutic candidates for further development.

## 2. Materials and methods

### 2.1. Materials, cell lines, and ethical statements

Recombinant human PD-L1-Fc, which comprises the extracellular domain of PD-L1, was purchased from PeproTech (Thermo Fisher, Cranbury, NJ, USA). Recombinant human PD-L1-His was previously produced in-house by Dr. Sibel Kalyoncu Uzunlar and Dr. Mehmet İnan. Complete and incomplete Freund’s adjuvants, PEG Hybri-Max, hypoxanthine-aminopterin-thymidine (HAT), hypoxanthine-thymidine (HT), antimouse IgG-HRP conjugate, antimouse IgG-AP conjugate, and streptavidin-HRP conjugate were obtained from Sigma-Aldrich (St. Louis, MO, USA). Antimouse IgG Fab2 Alexa Fluor-488 and 4′,6-diamidino-2-phenylindole (DAPI) were supplied by Cell Signaling Technology (Danver, MA, USA). The recombinant antigens used in cross-reactivity and inhibition assays were commercially obtained from BioLegend (San Diego, CA, USA), Sino Biological (Beijing, China), and BPS Biosciences (San Diego, CA, USA). The luciferase reporter PD-1/PD-L1 blockade bioassay was provided by Promega (Madison, WI, USA). Atezolizumab and avelumab were obtained from Selleckchem (Houston, TX, USA). The murine myeloma F0 cell line (ATCC, Manassas, VA, USA) and the human cancer cell lines MDA-MB-231 and A549 (both from ATCC) were cultured in high-glucose DMEM, and NCI-H292 and PC-9 cells (both from iCell Biosciences, Shangai, China) in RPMI-1640 medium supplemented with 10% FBS, 1% nonessential amino acids, and penicillin and streptomycin antibiotics (100 U/mL each) were cultured in a humidified incubator at 5% CO_2_. All cell lines were routinely tested for mycoplasma contamination (e-Myco, iNtRON Bio, Seongnam, South Korea). All protocols related to the housing, care, and generation of hybridomas were conducted in accordance with institutional guidelines and were approved by the Local Animal Ethics Committee of Gebze Technical University (Approval Number: 2020-02-03).

### 2.2. Monoclonal antibody production

Female BALB/c mice of 6 to 8 weeks of age were immunized with 50 μg of recombinant human PD-L1-Fc emulsified in complete Freund’s adjuvant, followed by three biweekly booster injections of 25 μg of antigen in incomplete Freund’s adjuvant. Serum antibody titers were monitored via indirect ELISA. The mice received a final prefusion boost of 25 μg of PD-L1. Three days later, isolated splenocytes were fused with murine F0 myeloma cells at a 5:1 ratio using PEG Hybri-Max. Fused cells were cultured in 96-well plates precoated with BALB/c feeder splenocytes in high-glucose DMEM supplemented with 1X HAT and 20% FBS. Emerging hybridoma colonies were screened for anti-PD-L1 reactivity by ELISA. Positive clones were subcloned in high-glucose DMEM supplemented with 1X HT medium at least twice by limiting dilution.

### 2.3. ELISA

ELISA plates (MaxiSorp, Nunc, Thermo Fisher Scientific, Waltham, MA, USA) were coated overnight with 1 μg/mL of recombinant human PD-L1-Fc per well. The following day, plates were washed with TBS-T buffer (Tween-20; 0.05%) and blocked with 5% nonfat skimmed milk in TBS-T. After washing, hybridoma supernatants were applied and incubated for 1 h at room temperature. Each sample was tested in duplicate, with immune mouse serum and hybridoma growth medium acting as the positive and negative controls, respectively. After washing, antimouse IgG-AP conjugate was added at a dilution of 1:1000 for 1 h at room temperature. The colorimetric reaction was generated using pNPP substrate and the MultiSkan FC microplate reader (Thermo Fisher Scientific) was used to measure absorbance values at 405 nm.

### 2.4. Assessment of antibody specificity via Western blotting and immunofluorescence

Western blotting and immunofluorescence experiments were performed on selected clones as previously described (Odabas et al., 2018; Güngör Topcu et al., 2025). Sixteen clones were analyzed using Western blotting on the MDA-MB-231 and the A549 cell lines. Cell lysate (50 μg) was run on 10% SDS-PAGE gel and transferred to a nitrocellulose membrane. After blocking with 5% nonfat dry milk, membranes were treated with anti-PD-L1 hybridoma supernatant diluted in blocking buffer at a ratio of 1:1. HRP-conjugated secondary antibody (1/5000, Advansta, San Jose, CA, USA) and WesternBright ECL (Advansta) chemiluminescence substrate were used to visualize protein bands with the ChemiDoc XRS system (Bio-Rad, Hercules, CA, USA). For immunofluorescence imaging, NSCLC cell lines NCI-H292 and PC-9 were processed using 14A8 and 15F6 hybridoma supernatants and a secondary antibody (antimouse IgG Fab2 Alexa Fluor 488) diluted 1:600 in 1% BSA-PBS. DAPI was used for nuclear staining at a dilution of 1:2000 in PBS. Slides were visualized using the LSM 800 Airyscan confocal microscope (Zeiss, Oberkochen, Germany).

### 2.5. Determination of antibody specificity

The determination of antibody isotypes was accomplished using the Rapid ELISA Mouse mAb Isotyping Kit (Thermo Fisher Scientific). Hybridoma supernatants were applied as the primary antibody and the experiment was completed according to the manufacturer’s instructions.

### 2.6. Cross-reactivity control

The cross-reactivity of mAbs with human immune checkpoint proteins (PD-L2, CD80, CD86, PD-1, CD28, TIGIT, TIM3, and VISTA) and PD-L1 orthologs (in mouse, rat, cynomolgus monkey, and canine species) was assessed using ELISA. Briefly, plates were coated with 100 ng of antigens and the reactivity of the hybridoma supernatants was quantified at 405 nm.

### 2.7. Evaluation of receptor blocking potential by competitive ELISA

The ELISA plates were coated with either 100 ng of PD-1 or 250 ng of CD80 per well overnight at 4 °C. Following standard blocking, the plates were washed three times with 1X PBS-T. For the PD-1/PD-L1 competition assay, 100 ng of biotinylated PD-L1 and 200 ng of anti-PD-L1 mAbs, and for the CD80/PD-L1 competition experiment, 250 ng of biotinylated PD-L1 and PD-L1 mAbs were added to the wells simultaneously and incubated for 90 min. After washing, streptavidin-HRP (1:1000, Merck, Darmstadt, Germany) was applied and the plate was incubated for 1 h. The colorimetric reaction that developed using an ultrasensitive TMB substrate (Merck) was stopped after 20 min with 1 M sulfuric acid. The absorbance was then measured at 450 nm. A positive control was provided by wells containing only biotinylated PD-L1 without the antibodies. The percentage of inhibition was calculated using the following formula: 100 × [1 − (A_450_ of sample / A_450_ of positive control].

### 2.8. Expansion of hybridoma culture and purification of antibodies

The hybridoma cells were expanded using DMEM supplemented with 10% FBS and then transferred to a serum-free medium for 3–4 days. The supernatants were collected by centrifugation and validated for PD-L1 reactivity by ELISA. Purification was performed using protein A or protein G affinity chromatography depending on the mAb isotype. The supernatant was incubated with the matrix overnight at 4 °C and then loaded into a chromatography column. Following the washing of the column by PBS, the mAbs were eluted with a citrate buffer (0.1 M sodium citrate, pH 3.0) or glycine buffer (0.1 M glycine-HCl, pH 2.7) for the protein A and protein G column, respectively. The eluates were immediately buffered using 100 mM Tris buffer (pH 9.0). The fractions were screened by ELISA, and positive fractions were concentrated and transferred to sterile PBS by centrifugation at 3000 × *g* and 4 °C using Amicon Ultra-15 (30K) centrifugal filters (Merck). Final antibody concentrations were determined using a NanoDrop device (Thermo Fisher Scientific) and the BCA assay. The purified mAbs were run on SDS-PAGE gel under reducing conditions and stained with Coomassie Blue.

### 2.9. Determination of the affinity of PD-L1 monoclonal antibodies by SPR analysis

SPR analysis was performed with a Biacore T200 system (Cytiva, Marlborough, MA, USA). Briefly, recombinant PD-L1 was immobilized onto a Series S CM5 chip at target density of 500 RU with an amine-coupling kit. A fourfold dilution series ranging from 128 nM to 0.5 nM antibody was then prepared and run over both the PD-L1 immobilized and blank channels with an association period of 120 s and a dissociation period of 600 s. A glycine 2.0 regeneration step was included between each cycle. The signal was double-referenced to both 0 nM analyte and a blank immobilization channel. Kinetic fitting was performed with a 1:1 binding model using Biacore T200 evaluation software (Cytiva) and K_D_ values were obtained from these fits.

### 2.10. Sequence analysis of the variable regions of heavy and light chains of antibodies

Total RNA was isolated from anti-PD-L1 hybridoma cells using TRIzol (Thermo Fisher Scientific) and the cDNAs were synthesized using a First Strand cDNA Synthesis Kit (New England Biolabs, Ipswich, MA, USA). The variable heavy (VH) and variable light (VL) chain gene regions of the mouse antibodies were amplified separately using an IgG primer set specific for murine antibody variable regions (PROGEN, Heidelberg, Germany). The amplicons were separated by agarose gel electrophoresis, and DNA bands of approximately 400 bp were excised and purified using the Monarch DNA Gel Extraction Kit (New England Biolabs). These PCR products were then cloned into a linearized vector supplied with either the PCR Cloning Kit (New England Biolabs) or the CloneJET PCR Cloning Kit (Thermo Fisher Scientific) at a vector-to-insert ratio of 1:3. Two microliters of the total ligation reaction volume (10 μL) were transformed into NEB5α chemically competent *Escherichia coli* using the heat-shock method. The transformed bacteria were plated onto 2× TYG agar plates containing 150 μg/mL ampicillin and incubated overnight at 37 °C. Three or more colonies were selected for each cloning reaction and inoculated onto LB broth containing 150 μg/mL ampicillin. These cultures were then incubated overnight at 37 °C with shaking at 250 rpm. Plasmid DNA was isolated from these cultures using a Plasmid DNA Miniprep Kit (Bio Basic, Markham, ON, Canada). The purified plasmids were subsequently sent for Sanger sequencing by Macrogen (Seoul, South Korea). The obtained sequences were analyzed using SnapGene (Dotmatics, Boston, MA, USA), and the VH and VL protein sequences were determined.

### 2.11. Assessment of PD-1/PD-L1 blockade by cell-based luciferase assay

A cell-based luciferase reporter bioassay kit (Promega) was used to assess functional receptor blockade, with all samples tested in triplicate strictly following the manufacturer’s instructions. The 24C11 and 17F8 mAbs were tested in duplicate at nine different concentrations. Avelumab and atezolizumab were included as the reference standards, and a mouse IgG1 isotype control antibody was included as the negative control. Briefly, 100 μL of PD-L1+ aAPC/CHO-K1 cells were seeded into each well of a white, flat-bottomed 96-well plate and incubated overnight at 37 °C in a 5% CO_2_ atmosphere. After removing the medium, 40 μL of the test and reference antibodies, which had been prepared at a concentration of 2× in assay buffer in the range of 0.016 to 25 μg/mL, were added to the appropriate wells. PD-1 effector T cells were then prepared according to the kit’s instructions. Subsequently, 40 μL of the cell suspension was dispensed into each well, except those containing only assay buffer, which was used as the assay background. The plate was incubated at 37 °C at 5% CO_2_ for 6 h and the assay also included no antibody control wells. After incubation, the plate was cooled to room temperature for 5 min, after which 80 μL of Bio-Glo reagent (Promega) was added to each well. Following 10 min of incubation at room temperature, luminometric measurement was carried out using the Varioskan Flash Microplate Reader (Thermo Fisher Scientific). Fold induction was calculated using the following relative light unit (RLU) formula provided in the kit’s manual: RLU (antibody – background)/RLU (no antibody control – background).

### 2.12. Statistical analysis

Statistical analyses and data visualization were performed using GraphPad Prism software (version 8.0, GraphPad Software, San Diego, CA, USA). Data are presented as the mean ± standard deviation (SD) of replicates. Half-maximal inhibitory concentration (IC_50_) values were calculated using nonlinear regression curve fitting with a four-parameter logistic equation.

To evaluate the effects of the antibodies tested in the luciferase reporter assay, two-way analysis of variance (ANOVA) was employed, followed by the Dunnett multiple-comparison test. Values of p < 0.05 were considered statistically significant.

## 3. Results

### 3.1. Development and selection of novel anti-PD-L1 hybridoma clones

**Figure 1:**
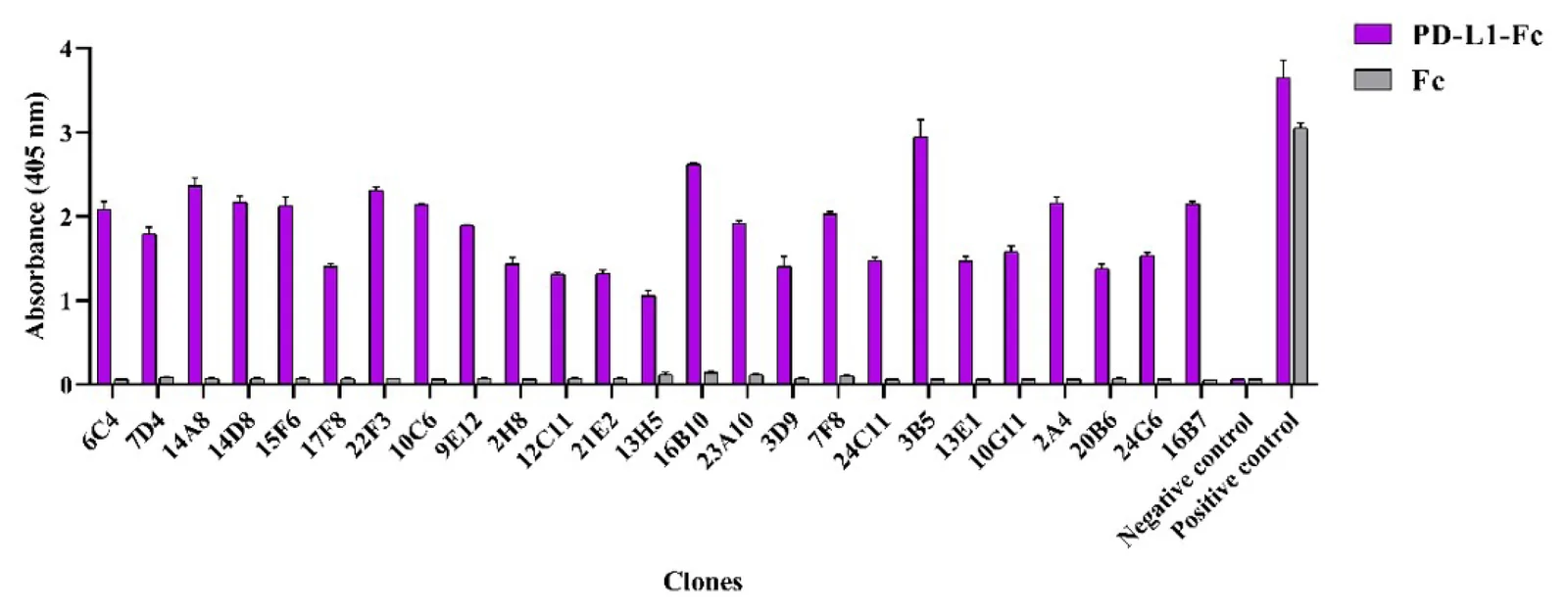
Development and selection of novel anti-PD-L1 hybridoma clones. The culture supernatant of the hybridomas was screened using ELISA. All clones reacted with PD-L1-Fc but not the Fc control antigen, demonstrating specificity for PD-L1. Hybridoma growth medium and mouse immune serum were included as negative and positive controls, respectively. The data are presented as the mean ± SD of three technical replicates.

Following the fusion process, the hybridomas were screened using ELISA against PD-L1 and Fc control antigens. Twenty-five PD-L1-specific clones were obtained and subjected to at least two rounds of subcloning. While the selected clones showed strong reactivity for rhPD-L1 (A_405_ > 1.0), binding to the Fc control did not reach levels higher than the negative control (A_405_ ≤ 0.15). This confirmed that the antibodies were specific to the extracellular domain of human PD-L1 (Figure 1). Isotype analysis revealed that all clones had kappa light chains, but the distribution of heavy chains varied. Fifteen clones were identified as IgG1, eight as IgG2a, and two as IgG2b (Table 1).

### 3.2. Assessment of the specificity of monoclonal antibodies for PD-L1

**Figure 2:**
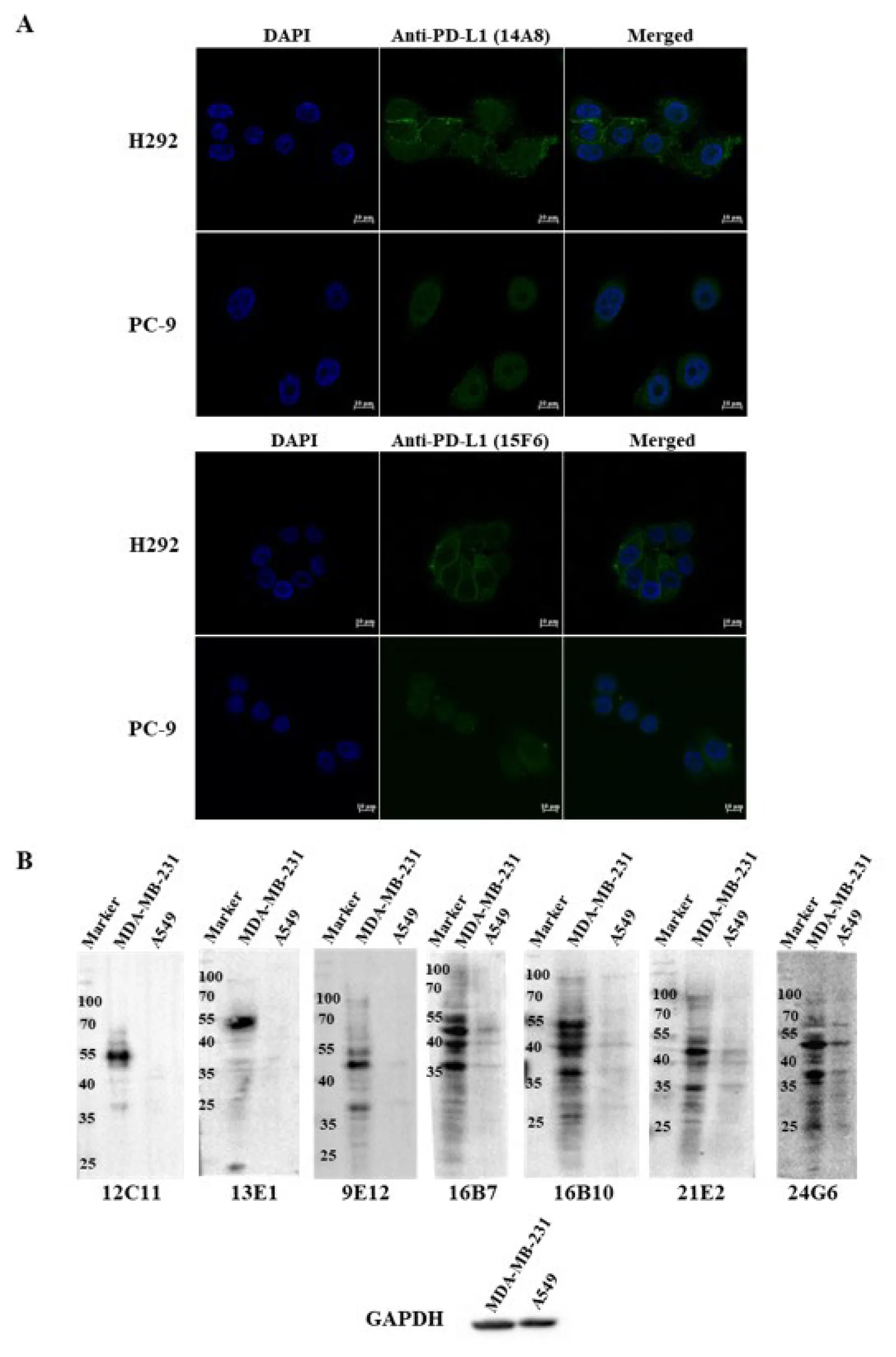
Specificity of the PD-L1 monoclonal antibodies was evaluated by immunofluorescence staining and Western blot analysis. A) Immunofluorescence staining was performed on NCI-H292 (PD-L1 high) and PC-9 (PD-L1 low) cells. PD-L1 was visualized using 14A8 and 15F6 antibodies (green), while the nuclei were stained with DAPI (blue). Merged images are shown on the right. Scale bars: 100 μm. B) Representative Western blot analysis of PD-L1 expression in MDA-MB-231 (PD-L1 high) and A549 (PD-L1 low) cell lines using the indicated monoclonal antibodies. The 12C11 and 13E1 antibodies selectively recognize glycosylated PD-L1. In contrast, 9E12, 16B7, 16B10, 21E2, and 24G6 clones exhibit a broad spectrum of reactivity. GAPDH was used to normalize the lysate loading across all antibody blots.

We selected PD-L1-positive NCI-H292 and PD-L1-negative PC-9 cells for immunofluorescence experiments and confirmed their PD-L1 expression profiles through flow cytometry and Western blotting analyses using commercial PD-L1 monoclonal antibodies (Figure S). First, the anti-PD-L1 monoclonal antibodies 14A8 and 15F6, which were initially generated, were examined using an immunofluorescence assay on PD-L1-positive and PD-L1-negative cells, respectively. Both antibodies exhibited specific membrane staining in the NCI-H292 cell line but not in the PC-9 cell line. This confirmed their reactivity with the extracellular portion of the PD-L1 protein (Figure 2A). The monoclonal antibodies were then subjected to Western blotting analysis using lysates from the MDA-MB-231 cell line, which expresses PD-L1, and the A549 cell line, which expresses low or no PD-L1 (Zheng et al., 2019). While most of the antibodies reacted with both the nascent (35 kDa) and the glycosylated (45–55 kDa) forms of PD-L1, only a few displayed selective binding to the glycosylated protein (Figure 2B).

### 3.3. Assessment of the specificity of monoclonal antibodies for PD-L1 across human checkpoint proteins and PD-L1 orthologs

**Figure 3:**
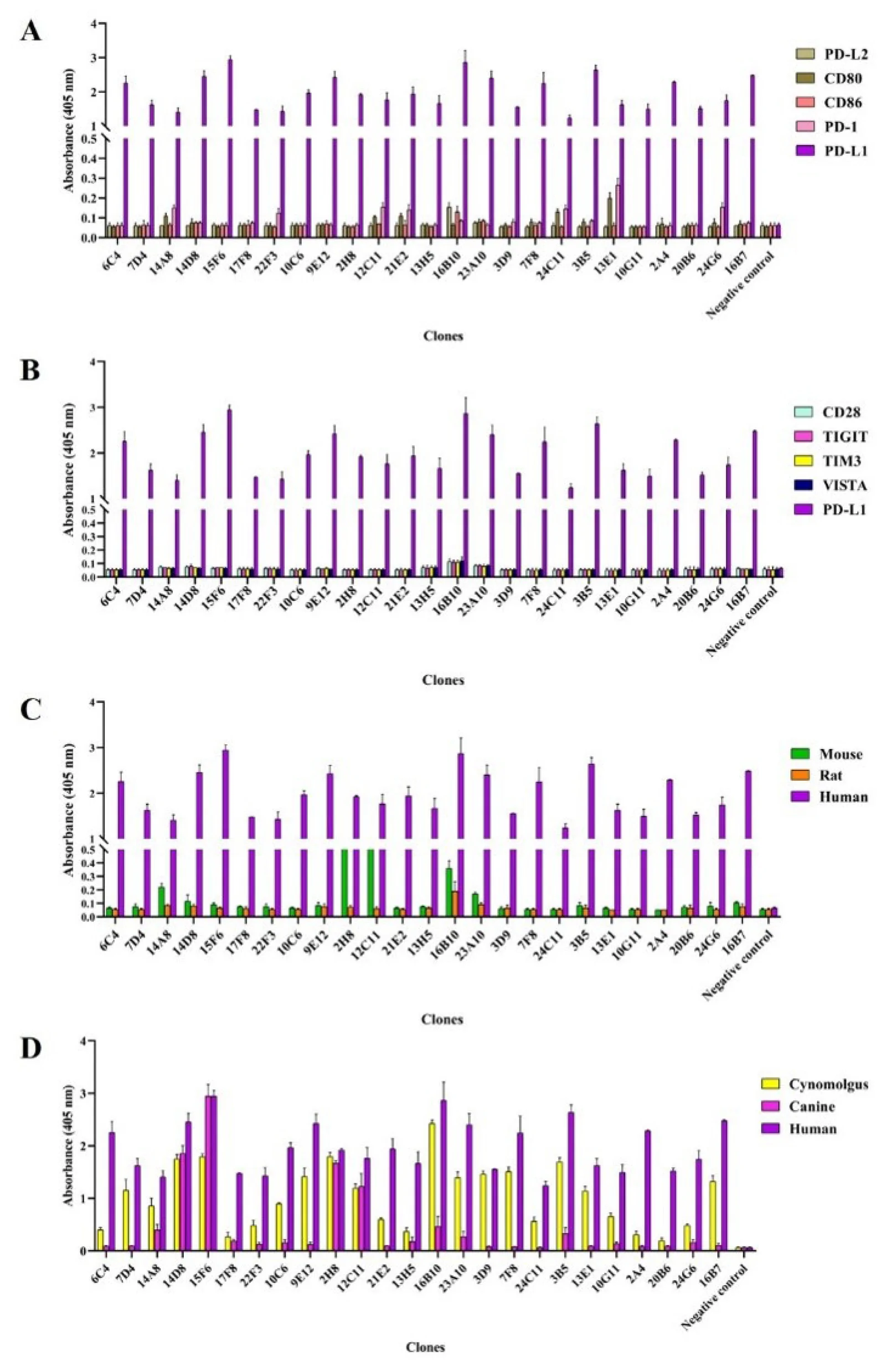
Analysis of the reactivity of monoclonal antibodies toward human immune checkpoint proteins and PD-L1 orthologs. A, B) ELISA-based screening of antibody binding using a panel of human immune checkpoint molecules to assess potential cross-reactivity. C, D) ELISA results show the reactivity profiles of the antibodies against PD-L1 orthologs from different species. Bars represent the mean absorbance values of the technical replicates.

To determine the specificity of the anti-PD-L1 mAbs, their cross-reactivity was evaluated via ELISA against a panel of human immune checkpoint proteins, including PD-L2, B7-1 (CD80), B7-2 (CD86), PD-1, CD28, TIGIT, TIM3, and VISTA. The generated clones demonstrated high specificity for PD-L1, showing no detectable binding to the other proteins in the panel (Figures 3A and 3B). The antibodies were also screened across PD-L1 orthologs (Figures 3C and 3D). Although the antibodies exhibited different levels of binding, the most notable cross-reactivity was observed with cynomolgus monkey PD-L1, followed by a subset of clones recognizing canine PD-L1. These differential binding profiles suggest that the generated clones target different epitopes on the PD-L1 molecule, some of which are conserved across species.

### 3.4. Evaluation of receptor blocking activity by competitive ELISA

Competitive ELISA was performed to evaluate the ability of affinity-purified PD-L1 mAbs to block PD-1/PD-L1 and CD80/PD-L1 interactions. Avelumab and atezolizumab were included as reference standards. The results showed that only three clones inhibited PD-1/PD-L1 binding, while 10 clones blocked CD80/PD-L1 interaction by more than 50% (Table 2). Of the clones examined for dual blockade potential, only 24C11 and 17F8 were considered promising drug candidates. Notably, 24C11 demonstrated inhibition capacity comparable to that of the reference antibodies.

**Figure 4:**
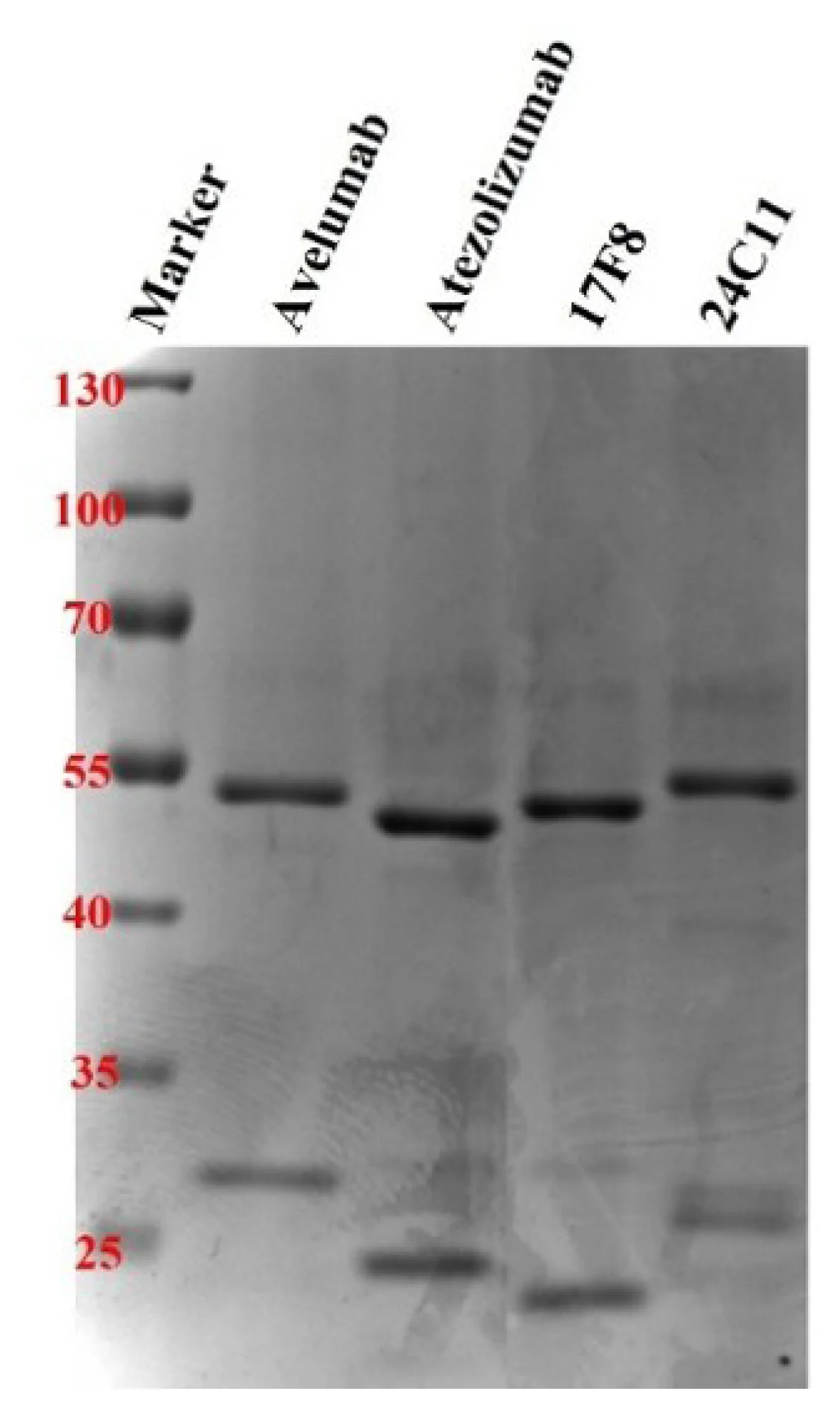
Assessment of antibody integrity and purity by SDS-PAGE. The 17F8 and 24C11 purified antibodies, together with reference antibodies avelumab and atezolizumab, were separated using SDS-PAGE under reducing conditions and stained with Coomassie Brilliant Blue R-250. The gel image illustrates the anticipated migration pattern of heavy (approximately 50 kDa) and light (approximately 25 kDa) antibody chains.

The integrity and purity of the 24C11 and 17F8 antibodies were assessed by examining them alongside avelumab and atezolizumab under reducing conditions using SDS-PAGE. Slight differences in the migration of heavy and light chains were observed. This heterogeneity in molecular weight among the different antibodies is consistent with variations in amino acid sequence length and differential posttranslational modification (Figure 4).

### 3.5. Determination of binding affinity against human PD-L1

SPR analysis was performed by running purified mAbs over a PD-L1-His antigen-coated CM5 chip (Table 3). The SPR data confirmed that clones 24C11 and 17F8, which have high blocking potential, displayed subnanomolar affinities for PD-L1. Clone 24C11 had a K_D_ value of 106.1 pM and a u-value of 2, while clone 17F8 had a K_D_ value of 422.2 pM and a u-value of 2. The u-value is a statistical parameter indicating the uniqueness of the fit parameters (rate constants and Rmax). Lower u-values signify better kinetic/affinity fit. In practice, u-values below 15 are considered acceptable for SPR analyses. In this study, the SPR results showed that both the 24C11 and 17F8 clones bind specifically to their PD-L1 target antigen with high affinity (in the pM range), as evidenced by high-quality fits to the experimental data.

### 3.6. Sequence analysis reveals the novelty of monoclonal antibodies 24C11 and 17F8

A competitive ELISA inhibition assay showed that both the 24C11 and 17F8 antibodies blocked PD-1/PD-L1 and PD-1/CD80 interactions, with the 24C11 antibody displaying higher blocking efficiency than the 17F8 antibody. SPR analysis, on the other hand, revealed that both antibodies exhibited subnanomolar affinity for PD-L1. Therefore, we selected clones 24C11 and 17F8 for the identification of their heavy and light chain variable sequences. The sequences of the CDR regions of these antibodies were then subjected to BLAST analysis, which revealed that the amino acid sequences of these monoclonal antibodies are unique (patent pending).

### 3.7. In vitro functional blockade of the PD-1/PD-L1 axis

**Figure 5:**
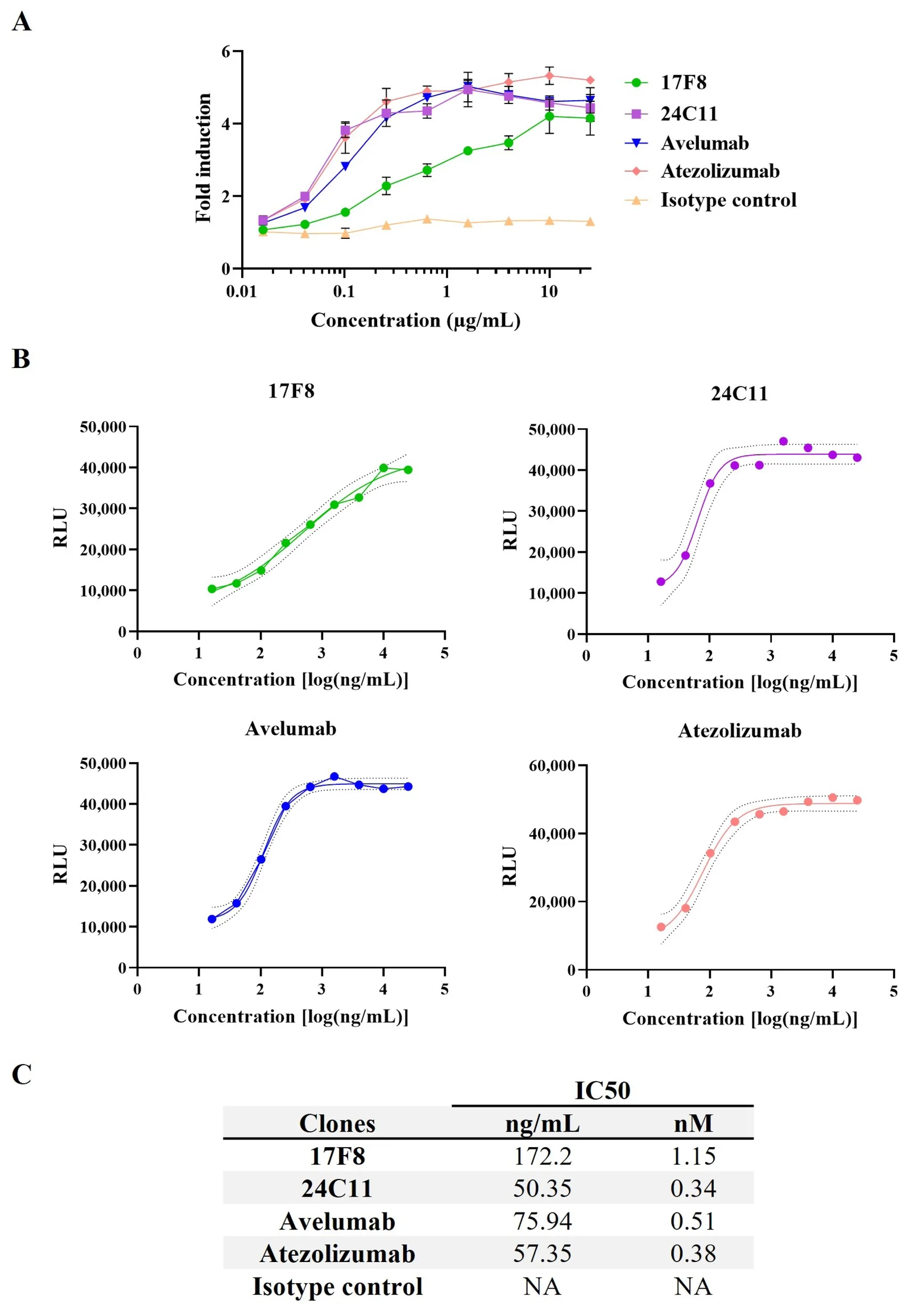
PD-1/PD-L1 cellular blockade assay. A) The blocking activity of monoclonal antibodies 17F8, 24C11, avelumab, and atezolizumab was determined at nine concentration points in a PD-1/PD-L1 reporter assay. Inhibition of PD-1/PD-L1 interaction was expressed as fold luminescence induction relative to the isotype control. Data represent the mean ± SD of technical replicates. B) Dose–response inhibition curves of the indicated antibodies. Luminescence values (RLU) were plotted against antibody concentration and sigmoidal curves were fitted to determine inhibitory activity. C) The calculated IC_50_ values for each antibody are expressed in ng/mL and nM. The isotype control showed no detectable inhibitory activity. NA: not applicable.

A cell-based luciferase reporter assay was performed to evaluate the ability of the antibodies to functionally disrupt PD-1/PD-L1 interaction in an engineered cell system expressing PD-1 and PD-L1 on the cell surface. The mAbs demonstrated a dose-dependent increase in the bioluminescent signal, indicating effective checkpoint blockade. Compared to the isotype control, none of the antibodies displayed significant induction at the lowest dose of 0.016 μg/mL. However, at a concentration of 0.041 μg/mL, the 24C11 antibody showed the most significant induction compared to the other antibodies (p < 0.0001). Starting at 0.256 μg/mL, all the antibodies exhibited similar neutralizing capacities at all the tested doses, except for 17F8. Overall, 24C11 was shown to have blocking potency as high as that of the reference antibodies (Figure 5A; Table 4). Data fitted using a four-parameter logistic regression model revealed a characteristic sigmoidal curve for 24C11, as was the case for atezolizumab and avelumab (Figure 5B). Notably, the half-maximal inhibitory concentration (IC_50_) values of our clones were in the nanomolar range (Figure 5C). In particular, the 24C11 antibody had efficacy equivalent or superior to that of the reference antibodies.

## 4. Discussion

Cancer therapy has been profoundly affected by immune checkpoint blockade, with better prognoses being provided for patients when it is administered either alone or in combination with conventional therapeutic modalities (Ribas and Wolchok, 2018). The use of monoclonal antibodies to target the PD-1/PD-L1 axis has become increasingly common in the treatment of various cancers, including NSCLC, stomach cancer, melanoma, urothelial cancer, and lymphoma (Bagchi et al., 2021). Atezolizumab, avelumab, durvalumab, and sugemalimab are among the recently approved PD-L1 therapeutic monoclonal antibodies, and several others are undergoing different phases of clinical trials (Xin et al., 2019). Accordingly, the objective of this study was to produce high-affinity PD-L1 monoclonal antibodies with the capacity to potently inhibit PD-1/PD-L1 interaction. Our rigorous screening strategy successfully identified 25 hybridoma clones that were highly specific to human PD-L1. This confirmed the effectiveness of the Fc counterselection approach that was used during the hybridoma selection process. Isotype analysis showed that the antibodies were predominantly of the IgG1 type, and to a lesser extent of the IgG2a and IgG2b types. This distribution is notable given that different antibody isotypes are known to confer distinct effector functions. It has been reported that murine IgG1 antibodies are generally associated with receptor-blocking activity, whereas murine IgG2a antibodies engage Fcγ receptors more efficiently and mediate antibody-dependent cellular cytotoxicity (Nimmerjahn and Ravetch, 2005; Dahan et al., 2015). The specificity of the antibodies to PD-L1 was clarified using immunofluorescence and Western blotting assays. Immunofluorescence imaging revealed clear membrane staining of selected cells by two different antibodies, confirming their reactivity with native PD-L1 under cellular conditions.

Further analysis using Western blotting revealed distinct binding patterns for the antibodies to PD-L1. While most of the antibodies reacted with multiple differentially glycosylated variants of PD-L1, only a few strictly recognized a band at approximately 55 kDa, which corresponds to the mature glycosylated form of PD-L1. This is consistent with previous reports showing that N-linked glycosylation shifts the molecular mass of PD-L1 from its precursor form at approximately 33 kDa, thereby contributing to the stabilization of immune evasion mechanisms (Li et al., 2016). Clones that can recognize this glycosylated form of PD-L1 are particularly valuable for detecting the biologically active receptor. Minor shifts in electrophoretic mobility observed in antibody chains during SDS-PAGE analysis were most likely due to differential glycosylation, contributing to the diversification of the antibody’s physicochemical characteristics (van de Bovenkamp et al., 2018).

The cross-reactivity observed with cynomolgus monkey PD-L1 for most of the antibodies is important for translational relevance. According to ICH S6 guidelines, cynomolgus monkeys are considered the standard relevant species for the preclinical safety assessment of therapeutic antibodies. Consequently, our antibodies’ ability to recognize cynomolgus PD-L1 suggests that they are suitable for future preclinical toxicology studies (Lu et al., 2020). Furthermore, the additional binding observed with canine PD-L1 indicates that these antibodies target evolutionarily conserved epitopes within the PD-L1 protein. The high specificity of the generated anti-PD-L1 antibodies is a critical safety feature. Notably, the absence of cross-reactivity with structurally related B7 family members such as PD-L2, B7-1, and B7-2 suggests that the biological activity of these antibodies is specifically mediated through blockade of the PD-1/PD-L1 axis. As B7/CD28 interactions are essential for T-cell costimulatory signaling, avoiding unintended binding to these ligands is important to prevent potential immunosuppressive or off-target immune effects (Sharpe and Freeman, 2002).

To prioritize therapeutically relevant candidates further, we implemented a stringent selection strategy that combined SPR-based affinity ranking with functional inhibition assays. Although strong binding affinity is necessary for effective target engagement, it is the ability to disrupt PD-1/PD-L1 and CD80/PD-L1 interactions that ultimately determines therapeutic efficacy. Consequently, only clones exhibiting high-affinity binding and potent neutralizing activity were considered as lead candidates. Targeting PD-L1 rather than PD-1 could offer further immunotherapeutic benefits. Under physiological conditions, PD-L1 on tumor cells can interact with B7-1 (CD80) on T cells to form heterodimers that sequester B7-1 and prevent it from interacting with the costimulatory receptor CD28. Therefore, antibodies that bind to PD-L1 and interfere with its interaction with B7-1 may not only block PD-1-mediated inhibitory signaling but also restore B7-1:CD28-mediated costimulation (Butte et al., 2007).

Although biochemical assays can confirm high-affinity target engagement, cell-based systems are essential for validating physiological neutralizing capacity. Further functional validation in cell-based luciferase reporter assays demonstrated that the generated antibodies effectively restored T-cell receptor signaling by blocking PD-1/PD-L1-mediated inhibition. Notably, clone 24C11 exhibited nanomolar IC_50_ values and a dose–response profile comparable to those of the clinically approved PD-L1 antibodies atezolizumab and avelumab. This supports its potential as a therapeutic candidate for restoring antitumor immune responses. Despite these promising findings, the limitations of the present study should be acknowledged. The current investigation primarily relied on biochemical and cell-based functional assays, with no in vivo validation included. Therefore, the therapeutic efficacy and safety profile of the lead antibody candidates must be evaluated using appropriate NSG or humanized mouse tumor xenograft models to accurately reflect the response of the human immune system (Chuprin et al., 2023). Furthermore, epitope mapping was not performed. Detailed structural characterization of the antibody–antigen interaction is important for defining the precise binding interface and could support the optimization of the therapeutic potential of these antibodies in future studies.

In conclusion, the present study has established a diverse panel of PD-L1-specific monoclonal antibodies that demonstrate strong functional blocking capacity and favorable specificity profiles. These findings provide a valuable basis for the ongoing development and optimization of next-generation therapeutic antibodies that target the PD-1/PD-L1 immune checkpoint axis.

## Supplementary information

### Assessment of PD-L1 expression in lung cancer cell lines

PD-L1 expression in the non-small cell lung cancer cell lines HCC827, NCI-H292, Calu-1, PC-9, and NCI-H520 was evaluated by Western blotting and flow cytometry. The Western blotting procedure is described in the main text. Western blot analysis demonstrated PD-L1 expression in the HCC827, NCI-H292, and Calu-1 cell lines, whereas low and undetectable expression levels were observed in PC-9 and NCI-H520 cells, respectively (Figure SA). To account for differences in protein loading, band intensities were quantified by densitometric analysis and normalized using Image Lab software (Bio-Rad). This analysis revealed that Calu-1 cells exhibited the highest relative PD-L1 expression among the cell lines examined.

Flow cytometry was performed to evaluate the cell surface expression of PD-L1 in lung cancer cell lines. Cells were cultured to subconfluency, washed with Ca^2_+_^- and Mg^2_+_^-free 1× PBS, and detached using Accutase. After counting, the cells were divided into three groups for staining: (i) unstained, (ii) PE-conjugated isotype control (BioLegend, catalog no. 400313), and (iii) PE-conjugated anti-PD-L1 antibody (BioLegend, catalog no. 329705). After centrifugation, the cell pellets were resuspended in 100 μL of cold PBS. Nonspecific binding was blocked by incubating the cells with 5 μL of Human FcR Blocking Reagent (Miltenyi Biotec) for 10 min in the dark. The cells were then incubated with the antibodies for 1 h at room temperature in the dark. After staining, the cells were washed with 900 μL of cold PBS and counterstained with DAPI. The final cell pellets were resuspended in 1 mL of cold PBS and analyzed within 1–2 h using an Accuri C6 flow cytometer (BD Biosciences). A total of 40,000 events were acquired for each sample. Flow cytometric analysis showed high PD-L1 expression in HCC827, NCI-H292, and Calu-1 cells, whereas low and undetectable expression levels were observed in PC-9 and NCI-H520 cells, respectively (Figure SB).

Figure S Assessment of PD-L1 expression in nonsmall cell lung carcinoma cell lines. A) PD-L1 expression was evaluated by Western blotting using an anti-PD-L1 antibody (GeneTex, catalog no. 104763) at a dilution of 1:4000. GAPDH (Advansta) was used as the loading control at a dilution of 1:5000. B) Cell surface PD-L1 expression was evaluated by flow cytometry. Cells were stained with either a PE-conjugated anti-PD-L1 antibody or the corresponding PE-conjugated isotype control antibody to assess surface PD-L1 expression.

## Figures and Tables

**Table 1:** Isotypes of PD-L1 monoclonal antibodies.

Clones	Heavy chain	Light chain
6C4	IgG1	Kappa
7D4	IgG1	Kappa
14A8	IgG1	Kappa
14D8	IgG1	Kappa
15F6	IgG2b	Kappa
17F8	IgG1	Kappa
22F3	IgG1	Kappa
10C6	IgG1	Kappa
9E12	IgG2a	Kappa
2H8	IgG2a	Kappa
12C11	IgG1	Kappa
21E2	IgG2a	Kappa
13H5	IgG1	Kappa
16B10	IgG1	Kappa
23A10	IgG2a	Kappa
3D9	IgG1	Kappa
7F8	IgG2a	Kappa
24C11	IgG1	Kappa
3B5	IgG2a	Kappa
13E1	IgG1	Kappa
10G11	IgG1	Kappa
2A4	IgG2a	Kappa
20B6	IgG2a	Kappa
24G6	IgG1	Kappa
16B7	IgG2b	Kappa

**Table 2:** Percentage inhibition of PD-1/PD-L1 and CD80/PD-L1 interactions by PD-L1 mAbs.

	% Inhibition	
Clone	PD-L1/PD-1	PD-L1/CD80
6C4	NA	NA
7D4	NA	NA
14A8	NA	94.4 ± 0.003
14D8	NA	NA
15F6	NA	NA
17F8	95.547 ± 1.552	48.52 ± 12.639
22F3	NA	70.41 ± 6.149
10C6	NA	NA
9E12	NA	52.26 ± 4.564
2H8	NA	NA
12C11	NA	NA
21E2	NA	72.16 ± 6.663
13H5	NA	94.5 ± 4.009
16B10	NA	24.24 ± 2.117
23A10	8.823 ± 0.067	0.77 ± 0.067
3D9	NA	NA
7F8	NA	62.1 ± 6.587
24C11	97.804 ± 1.736	93.25 ± 3.297
3B5	NA	NA
13E1	NA	52.12 ± 4.552
10G11	NA	51.28 ± 4.478
2A4	NA	14.99 ± 1.309
20B6	62.073 ± 6.240	NA
24G6	NA	NA
16B7	NA	88.88 ± 6.285
Avelumab	97.959 ± 3.532	93.69 ± 3.312
Atezolizumab	97.904 ± 1.092	94.43 ± 0.390

NA: not applicable. Data represent the mean values of technical replicates.

**Table 3:** SPR-based affinity analysis of selected hybridoma clones.

Clones	KD (pM)	u-value
6C4	8151	3
7D4	118	5
14A8	464	3
14D8	109	5
15F6	46.2	3
17F8	422.2	2
22F3	3567	2
10C6	840	4
9E12	68.3	9
2H8	1666	3
12C11	2167	3
21E2	94600	5
13H5	310.4	33
16B10	NA	NA
23A10	1529	9
3D9	124.9	7
7F8	2031	2
24C11	106.1	2
3B5	277	12
13E1	7600	7
10G11	1260	2
2A4	13400	7
20B6	221.7	3
24G6	836.2	4
16B7	119600	15
Avelumab	37.5	9
Atezolizumab	19	7

NA: not applicable.

**Table 4:** PD-1/PD-L1 cellular blockade assay.

	Fold induction				
Concentration (μg/mL)	17F8	24C11	Avelumab	Atezolizumab	Isotype
0.016	1.07 (ns)	1.33 (ns)	1.26 (ns)	1.33 (ns)	1.02
0.041	1.22 (ns)	2.00 (****)	1.68 (*)	1.92 (***)	0.97
0.102	1.56 (*)	3.82 (****)	2.82 (****)	3.62 (****)	0.98
0.256	2.28 (****)	4.29 (****)	4.16 (****)	4.62 (****)	1.20
0.64	2.72 (****)	4.35 (****)	4.72 (****)	4.90 (****)	1.37
1.6	3.25 (****)	4.94 (****)	5.03 (****)	4.91 (****)	1.26
4	3.47 (****)	4.76 (****)	4.79 (****)	5.15 (****)	1.32
10	4.20 (****)	4.57 (****)	4.61 (****)	5.32 (****)	1.33
25	4.15 (****)	4.44 (****)	4.64 (****)	5.20 (****)	1.30

* p < 0.05;

*** p < 0.001;

**** p < 0.0001;

ns: not significant.
